# Tai Chi for the treatment of chronic obstructive pulmonary disease: A systematic review protocol

**DOI:** 10.1097/MD.0000000000016097

**Published:** 2019-06-28

**Authors:** Longxia Gao, Dongxu Si, Haipeng Bao, MingXia Yu, Huizhuo Sun, Dashzeveg Damchaaperenlei, Yue Yan, Qi Shi, Youlin Li

**Affiliations:** a Beijing University of Chinese Medicine, Beijing; b Inner Mongolia Autonomous Region Hospital of Traditional Chinese Medicine, Hohhot; c The 2nd Department of Pulmonary Disease in TCM, The Key Unit of SATCM Pneumonopathy Chronic Cough and Dyspnea, Beijing Key Laboratory of Prevention and Treatment of Allergic Diseases with TCM (No. BZ0321), Center of Respiratory Medicine, China-Japan Friendship Hospital, National Clinical Research Center for Respiratory Diseases, Beijing, China.

**Keywords:** chronic obstructive pulmonary disease, protocol, systematic review, Tai Chi

## Abstract

**Background:**

Chronic obstructive pulmonary disease (COPD) is a common chronic respiratory disease with increasing morbidity and mortality that cause huge social and economic loss. Although recommended by guidelines, pulmonary rehabilitation has not been widely applied in clinics because of its inherent limitations. Free from restrictions of specific training venues and equipment, Tai Chi, as a kind of pulmonary rehabilitation, has been used to cure the COPD, yet the efficacy and safety of Tai Chi remains to be assessed. In this study, we aim to draw up a protocol for systematic review to evaluate the efficacy and safety of Tai Chi for COPD.

**Methods:**

We will search the following electronic databases from inception to December 31, 2018: PubMed, Web of Science, Medline, Cochrane Central Register of Controlled Trials, Springer, EMBASE, the China National Knowledge Infrastructure Database, Wan Fang Database, the Chinese Scientific Journal Database, and Chinese Biomedical Literature Database. Clinical trial registrations, potential gray literatures, relevant conference abstracts and reference list of identified studies will also be searched. The literature selection, data extraction, and quality assessment will be completed by 2 independent authors. Either the fixed-effects or random-effects model will be used for data synthesis based on the heterogeneity test. Changes in lung function will be evaluated as the primary outcome. Symptom assessment, quality of life (SGRQ), medication usage, exacerbations, and adverse events will be assessed as the secondary outcomes. The RevMan V.5.3.5 will be used for Meta-analysis.

**Results:**

This study will provide a synthesis of current evidence of Tai Chi for COPD from several aspects, such as lung function, SGRQ, medication usage, exacerbations, and adverse events.

**Conclusion:**

The conclusion of our study will provide updated evidence to judge whether Tai Chi is an effective solution to COPD patients.

**PROSPERO registration number:**

PROSPERO CRD42019122791.

## 1 Introduction

Chronic obstructive pulmonary disease (COPD) is a common, preventable and treatable disease with typical symptoms, including dyspnea, cough, and/or sputum production.^[1]^ Some patients also suffer from poor appetite, weight loss, peripheral skeletal muscle dysfunction. According to large-scale epidemiological surveys, it is estimated that the number of COPD cases in 2010 was 384 million, with a global morbidity of 11.7%.^[2]^ In China, the overall prevalence of spirometry-defined COPD was 8.6%, and the number increase to 13.7% among the general population aged 40 or older. The total number of individuals suffering from COPD was more than 100 million.^[3]^ Approximately 3 million die from COPD globally in 2015.^[4]^ With the increasing prevalence of smoking in developing countries and the aging populations in developed countries, the prevalence of COPD is expected to rise in the next 30 years. By 2030, more than 4.5 million people would die from COPD and relevant diseases every year.^[5]^ Besides, COPD patients may be subjected to respiratory infections, frequent exacerbations, and COPD-related hospitalizations, which contribute to a huge social and economic burden.^[6]^

COPD management aims to relieve symptoms, reduce the frequency and severity of exacerbations, and improve exercise tolerance and health status.^[1]^ Beta2-agonists, antimuscarinic drugs, methylxanthines, and inhaled corticosteroids (ICS) are major medications treating COPD. Symptoms can be controlled for most patients by using these drugs. However, there is no sufficient clinical trial evidence to prove that these drugs can prevent the long-term decline in lung function.^[7–10]^ As a Grade A Evidence, pulmonary rehabilitation has been recommended to COPD patients by the 2019 Chronic Obstructive Pulmonary Global Initiative. It has been proven to be the most effective treatment strategy to improve shortness of breath, exercise tolerance and health status.^[11]^ In addition, pulmonary rehabilitation may help reduce anxiety symptoms.^[1]^ Limited by geography, culture, finances, transport, and other logistics, the promotion and implementation of pulmonary rehabilitation faces many challenges.^[12–14]^

Rooted in Chinese traditional culture, Tai Chi is an exercise with distinctive Chinese characteristics, which consists of slow, continuous movements and provides mild to moderate aerobic activity.^[15,16]^ Furthermore, Tai Chi includes many aspects of modern medical pulmonary rehabilitation, such as lower limb exercise, upper limb exercise, respiratory training, nutritional support, and psychological adjustment. Tai Chi, as an enjoyable and relaxing sport, has a high adherence rate. It is not restricted by any specific training venues and equipment, ^[17,18]^ which makes it easy to be promoted in the communities and families.

Tai Chi exercise has also been applied as a training modality in pulmonary rehabilitation programs for COPD both in China and some western countries.^[19,20]^

In recent years, researches on Tai Chi for COPD increase, and research results are also diverse. Some preliminary evidence suggests that Tai Chi exercise may increase the body's muscle strength,^[21–23]^ enhance exercise capacity,^[24–26]^ improve lung function,^[25,18]^ and improve quality of life.^[27]^ However, some studies find that Tai Chi has no advantages in improving lung function, exercising tolerance and quality of life.^[17,28]^ The latest systematic review is uncertain about whether Tai Chi can enhance lung function and physical activity in COPD patients.^[29]^ Therefore, it is necessary to conduct a meta-analysis of Tai Chi for COPD. In this review, we aim to systematically evaluate the efficacy and safety of Tai Chi for curing COPD.

## 2 Methods

### 2.1 Study registration

The systematic review protocol has been registered on PROSPERO as CRD42019122791. Available from: http://www.crd.york.ac.uk/PROSPERO/display_record.php?ID=CRD42019122791

### 2.2 Inclusion criteria for study selection

#### 2.2.1 Types of studies

All relevant randomized controlled trials (RCTs) in English and Chinese will be included. Quasi-RCTs, cluster-randomized trials, duplicated publications, case report, and experimental studies will be excluded.

#### 2.2.2 Types of participants

Study participants in different age ranges with COPD can be included in the study without restricting nationality, sex, race, occupation, or education. Experimental objects include patients with chronic cough and wheezing caused by tuberculosis, tumors, irritating gas allergies, and other factors will be excluded.

#### 2.2.3 Types of interventions

The control group: basic treatment, including conventional medication and health education, patients in this group are not allowed to accept any form of lung rehabilitation during the study period. The experimental group: basic treatment and Tai Chi training. According to BTS Guideline on Pulmonary Rehabilitation in Adults, the physical activity will last for 6 to 8 weeks, 5 times a week for 30 minutes each time. Studies that do not meet the above criteria will be excluded.

#### 2.2.4 Types of outcome measures

##### 2.2.4.1 Primary outcomes

Changes in lung function (forced expiratory volume in the first second as a percentage of predicted value and first second forced expiratory volume occupancy lung capacity percentage will be assessed as primary outcome)

##### 2.2.4.2 Secondary outcomes

The secondary outcomes of this review will include:

1. Symptom assessment: COPD assessment test; breathlessness measurement using the modified British Medical Research Council; 6 minutes walk distance.
2. Quality of life (St. George's respiratory questionnaire).
3. Medication usage.
4. Exacerbations.
5. Adverse events.

### 2.3 Search methods for identification of studies

#### 2.3.1 Electronic searches

We will search the following electronic databases from inception to December 31, 2018: PubMed, Web of Science, Medline, Cochrane Central Register of Controlled Trials, Springer, EMBASE, the China National Knowledge Infrastructure Database, Wan fang database, the Chinese Scientific Journal Database, and Chinese Biomedical Literature Database. The search strategy for Medline (via PubMed) is shown in Table 1, other electronic databases will also be searched based on this strategy.

**Table 1:**
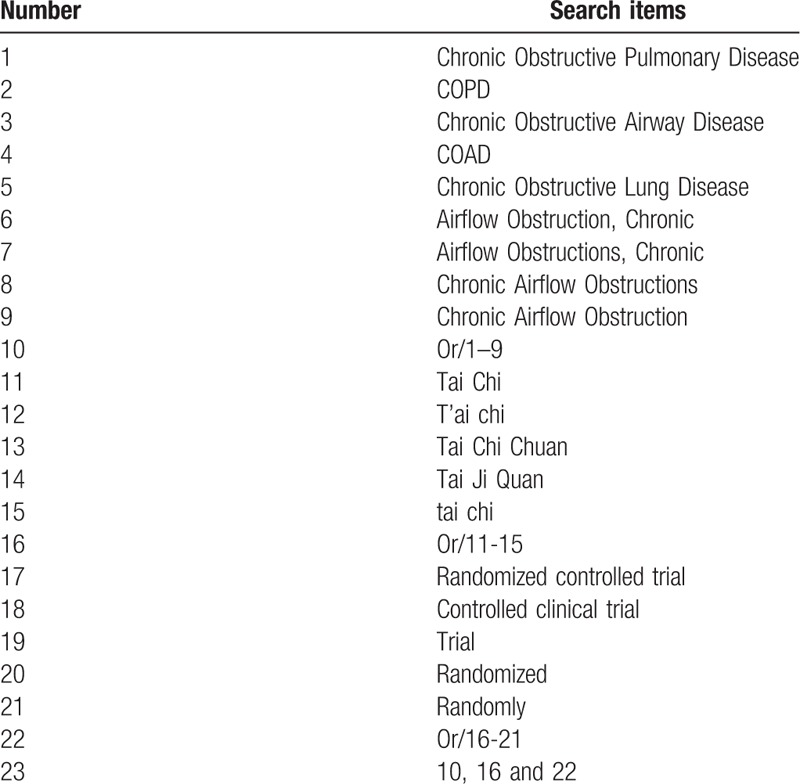
Search strategy for Medline (via PubMed).

#### 2.3.2 Searching other resources

The reference lists of studies and systematic reviews will be examined and retrieved for additional trials. Potential gray literatures will be searched in OpenGrey.eu. We will search relevant conference abstracts for eligible trials. In addition, we will also search the WHO International Clinical Trials Registry Platform and the ClinicalTrials.gov for all new reviews relevant to this topic.

### 2.4 Data collection and analysis

#### 2.4.1 Selection of studies

**Figure 1:**
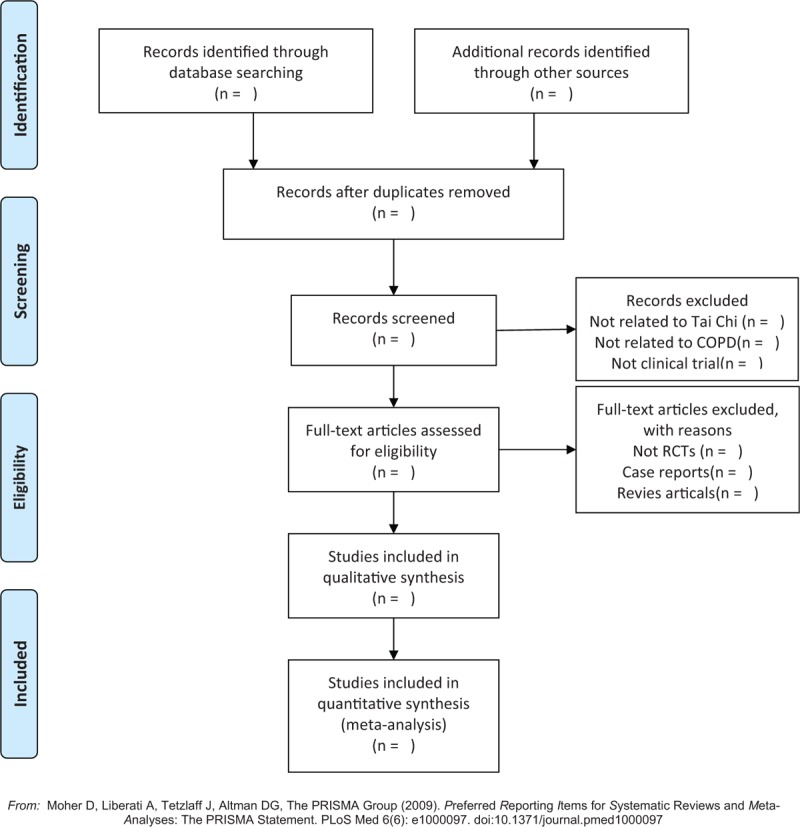
The PRISMA flow chart of the selection process. PRISMA = preferred reporting items for systematic reviews and meta-analyses.

We search the specified databases to obtain relevant literature, import them into a database created by Endnote X8 and screen out the duplicate documents. Two review authors will independently screen the titles, abstracts, and keywords of all retrieved records. If necessary, full texts will be examined according to the inclusion criteria for further assessment. We will resolve disagreements by discussing with the third author. The screening flow diagrams of this study will be shown in Figure 1.

#### 2.4.2 Data extraction and management

We will make a standard data collection sheet before data extraction. Two reviewers will independently extract data from the selected studies and fill in the data collection sheet. Discrepancies and uncertainties will be resolved by consensus between the 2 review authors or by asking the third author to make a final decision. We will extract the following data:

1. General information: the first author, title, the journal, publication type, publication year, country, fund source.
2. Methods: study design, sample size, randomization, allocation concealment, blinding methods, inclusion criteria, and exclusion criteria.
3. Participants: age, gender, severity of COPD, COPD diagnostic criteria, baseline lung function,
4. Interventions: type of control, duration of treatment, frequency of treatment.
5. Outcomes: primary and secondary outcomes, treatment costs, adverse effects, and follow up.

#### 2.4.3 Assessment of bias in the included studies

Two review authors will independently use the criteria outlined in the Cochrane Handbook for Systematic Reviews of Interventions to assess the risk of bias in the included studies. The following 6 domains in the Cochrane “Risk of bias tool” will be assessed: random sequence generation, allocation concealment, blinding, incomplete outcome data, selective reporting, and other bias. We will grade each potential trial of bias as high, low, and unclear. Any disagreement will be resolved by discussing or by asking the 3rd author to make a final decision.

#### 2.4.4 Measures of treatment effect

The continuous data will be expressed as mean difference (MD) or standard MD (SMD) with 95% confidence intervals (CIs), and the dichotomous outcomes will be estimated by the risk ratio (RR) with 95% CIs.

#### 2.4.5 Unit of analysis issues

In order to avoid carryover effects, we will only extract the first experimental period data of cross-over trials. For trials with multiple intervention groups, we will combine all relevant control intervention groups and experimental intervention groups of the trial into a single group to avoid a unit-of-analysis error.

#### 2.4.6 Management with missing data

For missing data, we will try to contact the original author to obtain the relevant data by email or phone. If we are unable to obtain missing data, the analysis will base on available data.

#### 2.4.7 Assessment of heterogeneity

Heterogeneity will be assessed by visual inspection of the forest plots and detected by standard Chi-squared test and *I*^2^ statistic. *I*^2^ <50% will be considered that the included studies are homogeneous, while *I*^2^ >50% will be taken as evidence of representing substantial heterogeneity. Sensitivity analysis and subgroup analysis will be selected to detect the possible reason of substantial heterogeneity.

#### 2.4.8 Assessment of reporting bias

When the number of included studies is more than 10, the funnel plot will be used to detect the reporting bias.

#### 2.4.9 Data synthesis

RevMan V.5.3.5 will be used for data analysis and synthesis. Continuous data will be expressed as MD/SMD with 95% CIs, while the dichotomous outcomes will be presented as RR with 95% CIs. When *I*^2^ <50%, the fixed effect model will be adopted to analyze. Otherwise, the random effect model will be selected. Additionally, we will use the sensitivity analysis and subgroup analysis to explore the causes of heterogeneity.

#### 2.4.10 Subgroup analysis

Subgroup analysis will be carried out based on the age of patients, different types of Tai Chi therapies, duration of treatment, frequency of treatment and the degree of asthma severity.

#### 2.4.11 Sensitivity analysis

If there are sufficient studies included, we will take sensitivity analyses to test the robustness and reliability of the results. The sensitivity analysis focuses on research characteristics or types such as methodological quality, and examines the effects of total effects by excluding certain low-quality studies or unblinded studies.

## 3 Discussion

At present, beta 2-agonists, antimuscarinic drugs, methylxanthines, and ICS are commonly used drugs for the treatment of COPD.^[1]^ However, both systemic and local side effects of them have been reported, such as hypokalemia, resting sinus tachycardia, ventricular arrhythmias, dryness of mouth, acute glaucoma, headaches, insomnia, steroid myopathy, oral candidiasis, hoarse voice, skin bruising, and pneumonia.^[30–34]^ What frustrates us is that many patients with COPD are still subjected to distressing breathlessness, impaired exercise capacity, fatigue, and suffer depression, anxiety, and panic, even when receiving optimal medical therapy.^[12]^ So, nonpharmacological interventions are urgently needed to alleviate clinical symptoms, reduce the risk of side effects and improve the quality of their life.

Based on the principles of traditional Chinese medicine theory-Yin and Yang and 5 elements, Tai Chi has been practiced for several centuries in China and has been gradually accepted by Western countries. Although the exact mechanism of Tai Chi treatment of diseases is still not fully clear, the results of clinical research show that Tai Chi has a certain effect on a variety of chronic diseases including COPD, and it is safe with no severe adverse effects.^[35–39]^ We hope this systematic review will provide more reliable evidence in the management of COPD.

There are still some limitations in this review. Limited by our language capability, we just collect studies in English and Chinese. Meanwhile, it is very difficult to use blind method in the process of Tai Chi treatment of COPD.

## Author contributions

LXG, DXS, and YLL proposed the concept of the study, developed the search strategy, LXG and DXS drafted the manuscript. QS and YY contributed to revise the manuscript and provided advice on the study. HZS and DD will assess the risk of bias and finish data synthesis. HPB will act the arbiter if disagreement occur. MXY and HPB will conduct statistical analysis. All authors read and approved the final manuscript.

**Conceptualization:** Longxia Gao, Dongxu Si, Youlin Li.

**Data curation:** Haipeng Bao, Huizhuo Sun, Dashzeveg Damchaaperenlei.

**Formal analysis:** Dongxu Si.

**Project administration:** Longxia Gao, Youlin Li.

**Supervision:** MingXia Yu, Yue Yan, Qi Shi.

**Writing – original draft:** Longxia Gao, Dongxu Si.

**Writing – review and editing:** Longxia Gao, Youlin Li.
